# Doctors experiences on the quality of care for pesticide poisoning patients in hospitals in Kampala, Uganda: a qualitative exploration using donabedian’s model

**DOI:** 10.1186/s12913-020-4891-6

**Published:** 2020-01-09

**Authors:** Charles Ssemugabo, Sarah Nalinya, Abdullah Ali Halage, Ruth Mubeezi Neebye, David Musoke, Erik Jørs

**Affiliations:** 1 Department of Disease Control and Environmental Health, School of Public Health, College of Health Sciences, Makerere University, P.O. Box 7072, Kampala, Uganda; 2 Department of Occupational and Environmental Medicine, Odense University Hospital, Sdr, Boulevard 29, DK-5000 Odense C, Denmark

**Keywords:** Pesticide poisoning management, Signs and symptoms, Structural, Processes, Outcomes

## Abstract

**Background:**

Pesticides are responsible for a significant percentage of deaths globally with majority occurring in sub-Saharan Africa. Deaths due to pesticide poisoning can be reduced if poisoning cases are managed optimally. However, the quality of care given to pesticide poisoning patients is still insufficient especially in sub-Saharan Africa. This study was aimed at exploring doctors’ experiences on quality of care for pesticide poisoning cases in hospitals in Kampala, Uganda.

**Methods:**

Fifteen (15) in-depth interviews were conducted with doctors who were directly involved in management of pesticide poisoning patients in the accident and emergency, Medicine, Pediatrics and Intensive Care Unit wards in 5 hospitals in Kampala, Uganda. All interviews were transcribed and subjected to directed content analysis with the guidance of the Donabedian model of quality of care which emphasizes structure, process and outcome measures as pertinent to ensuring quality of care.

**Results:**

Doctors reported structural, process and outcome facets that support diagnosis and treatment of pesticide poisoning cases that improved the quality of care they provided. Among the structures includes hospital units such as Intensive Care Unit (ICU), pediatrics and internal medicine; equipment and clinical guidelines such as airway, breathing and consciousness (ABC) protocol; and doctors’ knowledge and experiences. Doctors relied on history, and signs and symptoms to establish the cause and severity of pesticide poisoning. However, some patients and caretakers provided inaccurate pesticide poisoning history. Due to its availability in hospitals, doctors largely relied on atropine to manage pesticide poisoning cases whether or not relevant to treat the actual pesticide active ingredient responsible for the poisoning. Although majority of the cases treated recovered, those due to suicide were further referred to the hospital psychiatrist. Sharing experiences of managing pesticide poisoning patients among health workers and engaging in sensitization outreaches against pesticide poisoning were reported as potential activities to improve quality of care for pesticide poisoning patients.

**Conclusion:**

Doctors reflected on the structure, process and outcome measures of quality of care given to pesticide poisoning patients. The implications of hospital structures and clinical process to the quality of the outcomes of care demonstrates their importance in improving management of pesticide poisoning cases in hospitals in Kampala, Uganda.

## Background

Pesticide poisoning is a major public health problem in many low and middle income countries (LMICs) [1–3]. Although a global statistic is still not possible due to lack of large-scale rigorous surveillance data, it is estimated that over 7.4 million years of healthy life are lost to unintentional poisoning, a significant proportion of which is due to pesticides [4]. Pesticides which are used to kill or control pests including disease-carrying organisms and undesirable insects, animals and plants [5], are also responsible for over 3,000,000 hospitalised acute pesticide poisoning, 25,000,000 severe poisonings and 300,000 deaths per years [6].

Pesticide poisoning could be unintentional especially among farmers that frequently use pesticides or intentional. Unintentional/accidental poisoning usually occur among farmers and pesticide applicators [6]. Children are also affected when they get in contact with pesticides while playing [6]. Unintentional poisonings however account for a smaller percentage of deaths due to pesticide poisoning [7], with suicides accounting for majority of the deaths in sub-Saharan Africa [1, 7]. In fact, LMICs in sub-Saharan Africa are responsible for 3.5% of the global pesticides suicide deaths [1].

Management of pesticide poisonings involves immediate resuscitation including circulatory support and mechanical ventilation [8, 9]. Diagnosis is normally carried out through taking poisoning history and confirmed by clinical signs and symptoms, and decrease in cholinesterase [8]. However, in case of uncertainty of the diagnosis, a laboratory test using a suitable biomarker substance can be used to confirm the poisoning [8], Treatment is executed through administration of appropriate antidotes including atropine, oxime or benzodiazepine [10]. In some scenarios especially while using atropine, treatment might involve doubling the dose of the antidote until the poisoning victim improves [8]. These processes are very important in ensuring patients receive a good quality of care.

Quality of care for pesticide poisonings is a big problem especially in sub Saharan Africa. In LMICs especially sub-Saharan Africa, most health care workers have limited knowledge on the characteristics and management of pesticide poisoning [11]. In fact, majority of the health workers in sub-Saharan African countries are not familiar with the signs and symptoms of pesticide poisoning, their chemical groups, and the World Health Organization (WHO) pesticide classification categories [12]. The low levels of knowledge on management of acute pesticides poisoning could be attributed to limited opportunities to translate poisoning diagnosis and treatment guidelines into practice due to lack training on clinical guidelines and experience in managing poisoning cases [13] which compromises the quality of care.

Uganda is largely an agro-based country, with over 70% of its population practicing agriculture at small, medium and large/industrial level [14]. Majority of the farmers rely on pesticide to improve their yields. In fact, the amount of pesticides used in Uganda has increased from 338 tonnes in 1960 to 57,825 tonnes in 2016 [15]. The increase in use of pesticides is said to have resulted into increased occurrences of unintentional or intentional poisoning in Uganda. Despite presence of clinical guidelines on management of pesticide poisoning in Uganda [16], the quality of care for pesticide poisoning in hospitals where majority of the severe poisoning cases are referred is still a challenge and depends on several health system issues. Among them is infrastructure and medical supplies, and capacity of health workers [11]. In order to understand the quality of care received by pesticide poisoning patients, this study employed the Donabedian model of quality of care. In his model, Donabedian emphasizes structures, processes and outcomes of care as key aspects of quality of care [17]. It highlights that improvements in the structure of care results in improvements in clinical processes and consequently improvements in patient outcomes. Therefore, this study explored doctors’ experiences on quality of care for pesticide poisoning in hospitals in Kampala, Uganda.

## Methods

### Study setting

This was a qualitative study that involved interviewing of doctors in 5 out of the 28 public and private hospitals in Kampala the Capital City of Uganda. Kampala has a population of about 1,516,210 out of the country’s 34.8 million people [15]. The hospitals included Mulago, Mengo, Nsambya, International Hospital Kampala (IHK) and Nakasero hospitals. Mulago Hospital, with a bed capacity of 1790 patients, is a national referral hospital where patients are admitted from across the country, especially those severely poisoned, and patients do not pay user fees. Nsambya and Mengo are teaching and private not for profit hospitals with a bed capacity of 361 patients each. IHK and Nakasero hospitals with a bed capacity of 200 and 80 patients respectively are private for profit hospitals. Nsambya, Mengo, IHK and Nakasero hospitals are private thus charge user fees from patients. Within Uganda’s healthcare system, these hospitals are at the level of regional referral hospitals [18]. This study was carried out in hospitals in urban settings because most of the rural health centres refer pesticide poisoning patients to these health facilities due to lack of antidotes, and qualified health workers to manage these cases. In addition, these hospitals often referred pesticide poisoning patients to the Division of Government Analytical Laboratory (DGAL) for testing and confirmation of diagnosis.

### Study design and participants

This was a qualitative study that used in-depth interviews to collect data. Doctors who handle pesticide poisoning cases in wards where such cases are treated including the accident and emergency, internal medicine, pediatrics and Intensive Care Units (ICU) were purposively selected from the five participating hospitals. Of the approximately 24 doctors handling poisonings in the five hospitals, a total of 15 doctors – three per hospital were interviewed to the point of saturation. Of the 15 doctor, 5 were pediatrician, 5 were internal medicine consultants, and 5 were general doctors working on accidents and emergency units.

### Data collection

Qualitative data were collected using in-depth interview guides. The in-depth interview guide was designed based on reviewed literature [19–21] (Additional file 1). The guide explored doctors’ experiences on: 1) signs and symptoms of pesticide poisoning; 2) diagnosis and treatment of pesticide poisoning victims; and 3) positive and negative outcomes of management of pesticide poisoning patients. The in-depth interview guide was developed by a public health specialist (CS) and reviewed by Public health specialists (SN, AAH, RMN, DM and EJ) with over 15-year experience in conducting research before the final version was agreed upon. Prior to data collection, the tool was pre-tested among two doctors in a private hospital with similar setting as the study hospital that did not participate in this study. Necessary adjustments in the guide were made to ensure collection of quality data. Invitations to participate in the study were sent by public health specialist (SN) to selected hospitals and participants were provided with study information through the hospital. The interviews were conducted by public specialist (SN). The interviews were conducted from the hospitals (wards) where the doctors worked during day between 8 am and 3 pm. Each interview lasted 20 to 30 min. The interviews were audio-recorded.

### Data analysis

The interviews were transcribed verbatim by the public health specialist (SN). The transcripts were read by public health specialists (CS and SN) to develop a code book. The transcripts were imported into Atlas ti software version 7.0 and coded to organize data according to emerging issues. Qualitative data was analyzed using directed content analysis with the help of the Donabedian model of quality of care [17] as the guiding framework to identify recurrent themes and subthemes by categorizing the codes. Emerging codes are synthesized and sub-grouped into sub-themes. The sub-themes were then merged to form meaning units that are presented as themes. All themes are described in the results, and illustrated with selected quotes from participants.

### Ethical considerations

Ethical approval to conduct the study was obtained from the Makerere University School of Public Health Higher Degrees, Research and Ethics Committee (HDREC) protocol 322 and registered with the Uganda National Council of Science and Technology registration number 3947. Permission was also sought from the hospital directors to interview their staff. Written consent was obtained from doctors before conducting each interview. Identifiers instead of names of the doctors were used during the interviews to ensure confidentially.

## Results

The findings are organized and presented based on Donabedian model of quality of care. The findings show insights provided by the 15 doctors who participated in the study. The sample comprised of 5 females and 10 males from 2 private, 2 private not for profit and 1 public hospital. The doctors were from four departments; Accident and Emergency, Pediatrics, Medical and Intensive Care Unit wards.

### Structure of pesticide poisoning care

#### Hospital units

The doctors emphasized that the hospital units including wards such as the ICU at hospitals in which doctors deliver care to pesticide poisoning patients determined the quality of care they give to the patients. Presence of facilities such as enough hospital beds to admit the patients, intensive care equipment to support unstable patients and having the necessary medicines enabled the health care staff to save the lives of the pesticide poisoning patients they received.

One doctor stated:

*“Over 98% of the pesticide poisoning patients fully recover. It is because we [this hospital] have all the necessary equipment and manpower to save their lives. We have the ICU services, ventilation, enough medicines needed. Therefore, if the patient is admitted on time, we are most likely be able to save their life”* (Doctor 4, Medical ward).

Other doctors stated that even when the health care staff is well trained, limitations in physical settings such as insufficient equipment and medicine in the hospital constrained their ability to provide the necessary care to the pesticide poisoning patients.

*“You might have a patient; you know what to do but you don’t have the necessary equipment or medicines to treat the patient. In that case, I have major limitations and can’t do anything but refer the patient. We sometimes lose patients due to such avoidable reasons”* (Doctor 13, Medical ward).

The doctors also considered the presence of psychiatric units was an important factor in the quality of care offered to pesticide poisoning patients, especially those who were suicidal. All the doctors reported that their hospitals had a psychiatric unit where the patients who had taken pesticides for self-harm received further care to address the root cause of the poisoning.

“*All poisoning cases that are suicidal must be linked to the psychiatric unit before they’re discharged. If we don’t treat the root cause of the problem, some of them can go back home and find another way of committing suicide.*” (Doctor 2, Medical ward).

#### Hospital policies

All the doctors elaborated that their hospital policies required them to respond to pesticide poisoning cases as emergencies and admit them to monitor their condition, and the average duration of the hospital stays was between 1 day to 2 weeks depending on the severity of the condition in which the patients were received.

“*All cases of pesticide poisoning are treated as emergencies and even after they have stabilized, they must be admitted for at least a day or two as we monitor the signs and symptoms. It just applies as a general policy.”* (Doctor 7, Accidents and Emergency).

#### Health workers’ characteristics

The doctors emphasized the importance of clinical expertise in the quality of care provided to pesticide poisoning patients. Doctors elaborated that they used continuous professional development platforms such as mini-rounds, grand-round and seminars to share experiences and enhance their clinical expertise.

*“We hold these Continuous Medical Education (CME) sessions once a week to enable us to discuss interesting cases and share experiences with one another. We also have mini-rounds and grand-rounds, and these are also very important in sharing information. We also have seminars which can be held by different associations like the Medical Practitioners’ Association, Medical and Dental Council, Uganda Pediatric Association and others.”* (Doctor 3, Pediatrics Ward).

### Clinical processes for pesticide poisoning

#### Diagnosis

The doctors considered proper diagnosis as a critical first step in ensuring that pesticide poisoning cases receive the appropriate care needed. This was done through taking history from the patients or caregivers to determine the possible cause of poisoning, approximate time of poisoning, route of exposure, quantity of poison taken, and any first aid given to the patient. All these parameters enabled doctors decide on how to handle the patient.

*“When a patient is rushed in, our first concern is to determine the cause of illness. For most pesticide poisoning cases, we get all the history and circumstances of poisoning from the patient or caregiver. A proper history enables us make the right diagnosis and appropriate treatment plan for the patient.”* (Doctor 7, Accidents and Emergency).

Some doctors however noted that obtaining the proper history from the patients or caregivers was a challenge due to the panic and emergency surrounding pesticide poisoning patients. One of doctors said:

*“The challenge is that most of the caregivers cannot explain what the child took because they come rushing. It is quite difficult to treat the patient without enough history. Sometimes the caregivers lie about what happened and when you check the child, you find that it is something different.” (*Doctor 6, Pediatrics Ward*).*

The doctors said that they mainly depended on the signs and symptoms to determine whether a case was a pesticide poisoning patient. They emphasized that pesticide poisoning patients had a set of similar signs and symptoms which varied depending on the severity of the poisoning.

*“It’s quite easy to notice a pesticide poisoning patient and the signs and symptoms depend on the severity of the poisoning. Patients who had a slight dose come with a few signs like constricted pupils, nausea, vomiting, slight cardiovascular upsets, and diarrhoea but as severity progresses, we see drowsiness, drooling saliva and some become comatose. Very severe ones can get lingual spasms and bronchial spasms which cause de-circulation and hypertension”* (Doctor 1, Accidents and Emergency Ward).

The doctors said that physical and logistical challenges such as the lack of toxicology laboratories hindered them from producing detailed toxicology reports for the patients and only identified the general class of the pesticide poisoning agent.

“*Rarely do we go on to identify the specific kinds of pesticides or other poison, because our laboratories are not equipped to do such tests because they are very expensive.* (Doctor 5, Accidents and Emergency).

#### Treatment

The doctors emphasized the importance of standard guidelines and procedures in the proper treatment of pesticide poisoning patients. They identified the airway, breathing and circulation (ABC) protocol as the primary guidelines followed during treatment and most of these guidelines were pinned somewhere in the different wards where pesticide poisoning patients are treated.

*“When treating a case, we first look at the vital signs: The blood pressure, heart rate and whether the patient breathing. This is the ABC protocol. If the airway is okay, then we look at the breathing and then the circulation. When they stabilize and make sure that the ABC is fine, then we take the patient to ICU if it is necessary”* (Doctor 1, Accidents and Emergency).

For treatment anti-dotes, doctors said that they used atropine in the treatment of the pesticide poisoning cases. The frequency of administering atropine depended on the severity of the symptoms. However, it was the only anti-dote available in all hospitals.

*“The treatment procedure includes rehydrating the patient and then atropinisation follows. We do have an atropinisation protocol, we administer atropine to all our patients within 15minutes of diagnosis and then we continue after every 15-30minutes until total atropinisation has occurred and the pupils are fully dilated.”* (Doctor 6, Pediatrics ward).

### Patient and system outcomes

#### Recovery and rehabilitation

The doctors noted that most of the pesticide poisoning patients they handled, fully recovered and were discharged. This was mainly attributed to the presence of the necessary physical structures including medical and psychiatric wards and following the right procedures.

“*Most of our patient survive, get well and are discharged. I would say 95% of the pesticide poisoning cases are discharged unless they came in late with severe complications*. *We have adequate facilities to save lives*” (Doctor 7, Accidents and Emergency).

Some doctors elaborated that the positive relationships between the self-harm patients and the psychiatrists was essential for enabling patients to overcome negative emotions and fully recover.

“*We really recognize the need for psychiatric therapy. Most of the psychiatrists have a social work aspect and take time to understand and develop a positive relationship with the suicidal patients to enable them appreciate their self-worth and regain hope*” (Doctor 9, Medical ward).

#### Improving health workers’ competence

The doctors highlighted that sharing experience of managing various pesticide poisoning cases increased their competence. Sharing their experiences with other health workers also reminded them of the best practices that are necessary in managing complex pesticide poisoning cases.

*“Properly managing pesticide poisoning patients is very helpful because we keep learning and updating ourselves with new information. The junior members in the profession are also brought on board, we share experiences and it helps preserve the quality of the services offered.”* (Doctor 13, Medical ward).

#### Health promotion and wellbeing

An increase in the number of pesticide poisoning patients in the hospitals prompted doctors to conduct community sensitization to tackle the root causes, promote health and prevent pesticide poisoning.

“*As health workers, we feel the community has to know about pesticide poisoning because the source of poisoning is basically the community. We use the media and community outreaches to teach the community about poisoning, and how to handle it”* (Doctor 1, Accidents and Emergency ward)*.*

Some doctors added that the sensitization outreaches also led to better health care accessibility by the community. Having doctors and nurses in communities bridged the gap and improved the relationship between the health workers and the community.

“*The community sensitization that we carry out narrows the gap between the community and the health workers*. *The people get to understand that we can easily be approached in case they need help*.” (Doctor 5, Accidents and Emergency).

## Discussion

This qualitative study conducted among doctors working in the medical, ICU/accidents and emergency units and pediatrics ward in hospitals in Kampala provides insights into how structures and processes interact to improve health outcomes of pesticide poisoning patients. It reflects on the hospital units and policies in hospitals for management of pesticide poisoning, health workers’ characteristics, clinical processes and the patient and system outcomes of management of pesticide poisoning. The presence of sufficient and appropriate hospital equipment, standard policies and qualified health workers was considered important for ensuring quality of care for pesticide poisoning. Proper diagnosis and use of correct treatment procedures were critical steps in proper management of pesticide poisonings poisoning. Although most patients recovered, psychiatric therapy and health promotion activities were implemented to prevent more suicidal attempts and accidental poisoning. This demonstrates the need to invest more efforts in improving structures and processes in order to improve quality of care received by poisoning patients.

In our study, the doctors felt that the presence of appropriate hospital infrastructure such as intensive care units (ICU) or accident and emergency wards and equipment greatly enabled them to successfully manage pesticide poisoning cases. Presence of this infrastructure creates an enabling environment for health workers to provide quality healthcare. In fact, presence of adequate infrastructure builds confidence among health workers and patients [22]. Our findings are related to a study conducted in Vietnam where doctors were unable to provide quality care to the patients without appropriate clinical facilities and equipment [23]*.* Providing appropriate hospital infrastructure is key to enabling health workers’ optimal utilization of knowledge and experience to provide quality care to patients, thus reducing deaths due to pesticide poisoning.

From the results, doctors indicated that their clinical expertise and ability to make quick decisions were important in the proper management of pesticide poisoning cases. This finding is important because pesticide poisoning cases are emergencies, and timely initial treatment decisions are pivotal to save lives or prevent further complications [9]. These findings differ from those from a study carried out among health care professionals in Tanzania where majority did not have the adequate clinical expertise to manage pesticide poisoning patients [12]. The difference in the findings is probably due to the fact that our study was carried out among doctors while the Tanzanian study used a mix of physicians, clinical officers, and nurse practitioners that have limited expertise in management of pesticide poisoning patients. This implies that health facilities should prioritize regular enhancement of all health workers’ clinical expertise in management of pesticide poisoning through continuous professional development to enable them efficiently respond to rapidly changing circumstances.

Pesticide poisoning management policies and guidelines such as compulsory admission and the ABC protocol play a significant role in the delivery of quality care to pesticide poisoning patients as established in our study. Guidelines and policies provide clarity and re-affirm health workers’ roles and responsibilities especially in emergency situations which require quick decisions. An observational study in Sri Lanka and India was used to develop pesticide poisoning protocols [13], however, their use in most developing countries is still limited. For example, Uganda has clinical guidelines for management of pesticide poisoning [16], but health workers have reported no or limited use of these guidelines in management of poisoning cases. Therefore, health workers should be trained in use of clinical guidelines for diagnosis and treatment of pesticide poisoning cases.

Findings from our study show that the doctors depend on the history taken from patients or care givers and signs and symptoms to determine the cause and severity of the poisoning. This finding is understandable because pesticide poisoning patients usually present with a clear set signs and symptoms which can be used to make a diagnosis [9]. However, our study revealed that patients sometimes provide inaccurate history of the poisoning and this reduces the reliability of the diagnosis. Although history-taking and signs and symptoms may be less reliable than toxicology laboratory tests, evidence shows that pesticide poisoning is majorly diagnosed through history and signs and symptom [24–26]. Some scholars argue that with standardized case-definition and diagnosis tools, toxicology laboratory confirmation is unnecessary [27]. Therefore, frameworks for taking history and classifying pesticide poisoning symptoms are important in improving the reliability and quality of the diagnosis and treatment of pesticide poisoning cases.

Our study revealed that doctors treat pesticide poisoning cases using atropine as the mainstay anti-dote. Atropine is the most readily available antidote in all hospitals in Kampala, Uganda. In addition, atropine is one of the most important antidotes for treating / managing organophosphate poisonings [9, 10, 28]. In fact, majority of the pesticide poisoning in Uganda is attribute to organophosphates [29, 30]. Our findings are similar to studies carried out in Netherlands and Sri Lanka where organophosphates were largely found to be responsible for majority of the poisoning [8, 13]. This implies that essential medicines and medical supplies should always be available in health centres based on the common pesticide active ingredients that cause poisoning to facilitate proper treatment and management of pesticide poisoning cases.

Although most of the patients recovered, those due to self-harm were further referred to psychiatric units. This finding is important because psychiatric follow up enables health workers to diagnose underlying causes of self-harm such as depression and other mental disorders [29, 31]. Literature shows that self-harm patients account for the highest percentage of morbidity and mortality due to pesticide poisoning [32, 33]. This implies that efforts should be made to include psychiatric units in all health facilities handling pesticide poisoning patients. In addition, psychiatrists should develop positive relationships with patients and follow them up to ensure complete rehabilitation. Therefore, psychiatric treatment should be included in the Uganda clinical guidelines for management of pesticide poisoning cases due to suicide.

Our study also found that doctors engage in community prevention strategies such as health education as a result of receiving several cases of pesticide poisoning. This finding shows that management of pesticide poisoning patients also results into community health intervention geared towards prevention of poisonings. Evidence has shown that comprehensive health promotion and education interventions reduce the risk of pesticide poisoning [19, 34, 35]. On the contrary, community interventions such as the use of lockable pesticide storage at household level were found to have no effect on occurrence of pesticide poisoning [36]. The lack of difference in the control and interventions groups can be attributed to the study population of 14-years and above who can ably open lockable pesticide storages facilities. Therefore, while deciding on community interventions that can effectively reduce pesticide poisoning, it is important to understand the target population.

Our study provides key findings on structural, process and outcome measures of quality of care for poisoning patients. However, it had some limitations. Although according to Pederson et al., more pesticide poisoning cases occur in urban than rural areas [30], our study was carried out in hospitals in Kampala the capital city of Uganda that are highly resourced and served by qualified staff compared the rural health facilities. So, it is likely that many opinions of frontline health workers that handle poisoning cases in rural health facilities on a regular basis were missed. As such, our findings might not be transferable to rural health facilities. The study did not assess doctor’s actual knowledge of different classes of pesticides and the different treatments that are given according to the class of pesticide that has caused the poisoning.

## Conclusion

Presence of hospital units, and policies as well as health workers’ characteristics greatly influenced the quality of care for pesticide poisoning patients. Proper clinical processes such diagnosis and treatment also improved the quality of care. Despite recovery, rehabilitation was recommended for a couple of patients to ensure quality. Other system outcomes included improvement in health workers’ competences and health promotional campaigns. The effect of structure and process measures to outcome measures of quality of care demonstrates their importance to improving the quality of treatment to pesticide poisoning cases in hospitals in Kampala, Uganda.

## Supplementary information

**Additional file 1.** Health worker’s knowledge, diagnosis and treatment of acute pesticide poisoning cases.

## Data Availability

The dataset used during the study is available from the corresponding author on reasonable request.

## Abbreviations

ABC: Airway, breathing and circulation
CME: Continuous medical education
CPD: Continuous professional development
DGAL: Directorate of government analytical laboratories
HDREC: Higher degree and research ethics committee
ICU: Intensive care unit
IHK: International hospital Kampala
LMICs: Low and middle income countries
WHO: World Health Organization
