# Supplementary material for: Doctors experiences on the quality of care for pesticide poisoning patients in hospitals in Kampala, Uganda: a qualitative exploration using donabedian’s model
Source: BMC Health Serv Res. 2020 Jan 9;20:30. doi: 10.1186/s12913-020-4891-6 (PMC6953287; doi:10.1186/s12913-020-4891-6)
Supplement: Supplementary file 1 — Additional file 1. Health worker’s knowledge, diagnosis and treatment of acute pesticide poisoning cases. [file 12913_2020_4891_MOESM1_ESM.docx]

**Health Worker’s Knowledge Diagnosis and Treatment of Acute Pesticide Poisoning Cases**

**IN-DEPTH INTERVIEW GUIDE**

1. How would you describe the magnitude of pesticide poisoning in this ward

**Probe:** How many cases do you receive in a month, how severe are the cases, what are the common pesticides responsible for poisoning and what is the common outcome of the poisoning.

1. Do you have a diagnosis and treatment protocol for managing pesticide poisoning cases in this ward

**Probe:** What are some of the important procedures or guidelines that you follow to diagnose and give treatment?

1. Do you know the different types of pesticides that are responsible for most of the poisoning in this ward?

**Probe:** Briefly tell me about each of them, how toxic is each one of them and what are the common symptoms associated with each of the pesticides

1. How would you describe your success in treating/managing pesticide poisoning cases in this ward

**Probe:** what are the come outcomes and why?

1. What are some of the challenges you face in managing pesticide poisoning cases

**Probe**: How do you relate with patients, type of patients

1. How do you share individual experiences in managing a pesticide poisoning case in this ward?

**Probe:** Are there platforms for sharing experiences, mention them, and has sharing of experiences caused any improvement in the management of pesticide poisoning cases.

1. What are some of the potential opportunities in your practice that can be used to improve management of pesticide poisoning?
